# Customizable High-Throughput Chemical Phenotyping of Root Bacteria

**DOI:** 10.21769/BioProtoc.5710

**Published:** 2026-06-05

**Authors:** Lisa Thoenen, Caitlin Giroud, Claudia Probst, Liza Rouyer, Niklas Schandry, Klaus Schlaeppi

**Affiliations:** 1 Department of Environmental Sciences, University of Basel, Basel, Switzerland; 2 LMU Biocenter, Ludwig-Maximilians-University Munich, Martinsried, Germany

**Keywords:** Bacteria phenotyping, Root microbiome, Bacterial isolates, Antimicrobials, High-throughput, Tolerance testing

## Abstract

Chemical phenotyping is a fundamental technique to study the metabolic properties or chemical sensitivities of bacteria. Traditional methods such as dilution methods, discs, or gradient diffusion assays are labor-intensive, often have high material requirements, and are limited in scalability. High-throughput cultivation approaches based on 96-well plates scale efficiently to large numbers of samples. A stacker, when coupled with a plate reader system (often already available in most laboratories), greatly enhances assay scalability and robustness. Here, we describe a customized high-throughput, flexible, scalable, robust, and affordable method for the chemical phenotyping of bacteria. This liquid culture–based growth system allows screening many bacteria in parallel and in a replicated manner for their tolerance to various chemicals, including specialized metabolites of plants, antibiotics, or pesticides. Compared to commercial solutions, our approach offers high flexibility in experimental conditions while keeping costs for consumables low.

Key features

• Approach to determine tolerance of bacteria against diverse chemicals, including specialized plant metabolites.

• Experimental platform where parameters like strains, media, chemicals, concentrations, or exposure time can be flexibly varied for bacterial phenotyping.

• Coupling a stacker to a plate reader permits highly replicated and efficient screenings of large bacterial collections and numerous different compounds.

**This protocol is used in:**

bioRxiv (2021), DOI: 10.1101/2021.01.12.425818

PNAS (2023), DOI: 10.1073/pnas.2310134120

Nature Communication (2024), DOI: 0.1038/s41467-024-49643-w

bioRxiv (2025), DOI: 10.1101/2025.06.06.658260

mSphere (2025), DOI: 10.1128/msphere.00159-25

## Graphical overview


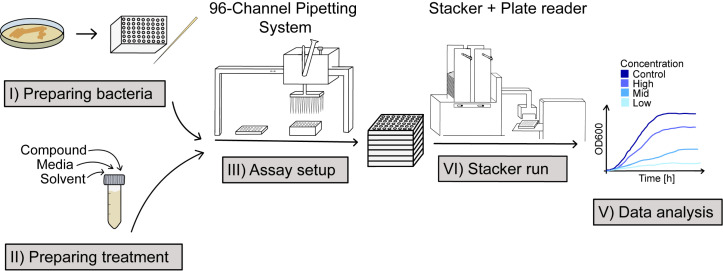


**High-throughput phenotyping of bacteria.** The scheme illustrates the five steps of the customizable high-throughput approach for chemical phenotyping of bacteria. (I) Preparation of bacteria from agar plates to liquid precultures. (II) Preparation of treatment solutions by mixing the culture media with the compound dissolved in a solvent. (III) Setting up the assay by combining bacteria and treatment solutions in 96-well plates using a 96-channel pipetting system. (IV) Starting a stacker run for parallel measurements of bacterial growth based on optical density. (V) Analysis of bacterial growth curves in the statistical software R.

## Background

Many research questions in bacteriology, ranging from studying nutritional preferences (e.g., carbon substrates) to metabolic capacities or sensitivities against various compounds, including specialized metabolites from plants, antibiotics, or environmental chemicals, rely on chemical phenotyping, i.e., the quantitative assessment of bacterial growth during chemical exposure. When studying the plant root microbiome, chemical phenotyping is useful to dissect interactions of root microbes with plant root exudates. Root exudates are secreted to the surrounding soil and contain primary metabolites, on which microbes feed, as well as specialized metabolites with attractant and repellent properties [1–3]. These bioactive metabolites can affect bacterial behavior, such as motility, biofilm formation, and chemotaxis [4–8]. Studies on how specialized plant metabolites, such as coumarins or benzoxazinoids, contribute to the structure of root microbiomes are a prime area for chemical phenotyping [9–11].

Traditionally, bioactive compounds are tested using dilution methods, disc, gradient diffusion assays, or chromogenic media [12]. These methods are labor-intensive, have high material requirements, and need large amounts of compounds to be tested. Alternatively, commercially developed phenotyping arrays (e.g., www.biolog.com) permit screening and testing for metabolic capacities and other microbial properties. Such systems are based on 96-well plates, have much lower material requirements, and allow highly standardized growth and cross-referencing of results. One limitation is the restriction on the range of chemical compounds as offered by the company; thus, only broadly used and commercialized compounds can be tested. However, several specialized plant metabolites are not included in commercial phenotyping arrays or are not commercially available and need to be isolated and purified. A second limitation is linked to scalability and costs, particularly when a research question requires the chemical phenotyping of large sets of bacterial strains, such as established strain collections of the root microbiome [13–18]. In our research on how specialized plant metabolites affect the growth of root microbiome isolates, we sought to develop a custom approach satisfying the following requirements: (i) flexible and scalable, allowing simple variation in numbers of strains, compounds, and concentrations; (ii) adaptable to any dissolvable chemical compound, which is available in sufficient amounts and stable in the given conditions; (iii) high-throughput, where many reactions and replications can be run simultaneously for the production of robust and reproducible data; and finally, (iv) affordable for a small laboratory. Inspired by commercial solutions and previous work [19], we developed our custom approach for chemical phenotyping of bacteria fulfilling these requirements.

A few considerations regarding the necessary infrastructure need to be noted: plate readers are routinely used in molecular biology to measure absorbance, fluorescence, or luminescence in microtiter plates, and they are available in many laboratories. Plate readers are costly instruments (approximately 75,000 in our case), and moderate investments can upgrade the scalability and throughput of experiments tremendously. The key investment is a stacker that automatically handles stacks of plates (typically between 15 and 25 plates; investment ~25,000). A second investment is a benchtop 96-channel pipetting system (investment ~12,000) that greatly speeds up the setup of many plates for replication of strains, compounds, and concentrations. The pipetting system makes it possible to set up a standard phenotyping run in less than 3 h, and the stacker then handles up to 25 × 96-well plates autonomously. Such a standard phenotyping run can include up to 60 strains in 3 replicates that are tested in 8 different conditions (compounds, concentrations, and controls). With hourly measurements, such a run will generate 70,000 datapoints in a 48-h assay. Overall, integrating high-throughput pipetting and automated measurements with multiple plates with a stacker significantly expands the capabilities of the plate reader, thereby enhancing the efficiency and scope of bacterial chemical phenotyping.

Here, we describe a customizable, high-throughput phenotyping approach that measures bacterial growth in the presence of chemical compounds originally developed in [20]. The basic requirements are that the bacteria grow aerobically, that the chemical compound of interest is stable in these conditions, and that, if a compound is not soluble in water, the used solvent does not prevent bacterial growth. Mild toxicity of the solvent can be controlled using appropriate controls. As the stacker and plate reader are not sterile environments, the assays require media controls (no-bacteria controls, NBCs) to detect potential contamination during a run. Using NBCs and media control plates, we typically do not detect environmental contamination for up to 68 h of assay length; however, contamination may depend on the media used. Our approach includes five steps, as illustrated in the Graphical overview. Preparatory steps are needed for the assay, including (i) pre-cultivation of the bacteria and (ii) preparation of the treatments, i.e., the stocks of the chemical compounds to be tested. Then, the 96-channel pipetting system helps to (iii) set up the assay plates, where the bacteria and their culture medium containing the different concentrations of the test chemicals are assembled. Then, (iv) plates are loaded onto the stacker, which handles them for autonomous recording of the optical density of the bacterial cultures over time with the plate reader. Finally, (v) exported data are analyzed using the open-source software R. We describe our approach based on an exemplary stacker run, for which we provide template data and a corresponding R script for analysis. In this example, we studied the antimicrobial activity of a specialized maize metabolite on the growth of maize root bacteria. Below, we provide the detailed protocol, including required materials and methodological steps. In the notes, we discuss limitations of our approach and offer recommendations for enhancing its scalability, as well as explore potential applications beyond the experimental example provided.

## Materials and reagents

*Note: Prepare and store all biological materials and solutions at room temperature unless indicated otherwise. Follow all waste disposal regulations.*

**Biological materials**

1. Glycerol stocks of bacterial isolates (BIS) to be tested

*Note: We describe this protocol based on an exemplary set of 60 BIS. We routinely use this protocol to investigate isolates of plant root bacteria [11,20–23]. See General note 1 on the choice of bacteria that can be tested with this approach.*

**Reagents**

1. Tryptic soy broth (TSB) (Millipore, catalog number: 22092) and agar to prepare solid and liquid media for bacterial cultivation; see General note 2 on the choice of media other than TSB that can be used with this approach

2. Stock solutions of the chemicals to be tested

3. Solvent for the chemicals to be tested

*Note: We describe this protocol based on an exemplary compound. However, we routinely use this protocol to investigate specialized plant metabolites like 6-Methoxy-2-benzoxazolinone (MBOA) (Sigma-Aldrich, catalog number: 543551) dissolved in dimethyl sulfoxide (DMSO) (Sigma-Aldrich, catalog number: D2438). See General note 3 on the choice of chemicals (and their solvents) to be used with this approach.*

**Laboratory supplies**

1. Sterile Ø 9 cm Petri dishes (Sarstedt, catalog number: 82.1473.001)

2. 50 mL centrifugation tubes (Greiner, catalog number: 227261)

3. Sterile filter tips for pipettes (20–200 μL) (Sarstedt, catalog number: 70.3031.355) or for the benchtop 96-channel pipetting system (Mettler Toledo, model: Liquidator 96^TM^, catalog number: 17010646)

4. Sterile filter tips for 8-channel pipette (100–1,000 μL) (Sarstedt, catalog number: 70.3060.355)

5. Sterile reagent reservoirs (25 mL for 8-channel pipette) (ThermoFisher, catalog number: 8094)

6. Deep round-bottom 96-well plates, 2 mL volume (Carl Roth, catalog number: EN07.1)

7. Sterile inoculation loops (VWR, catalog number: 612-7274) and needles (Greiner, catalog number: 731185)

8. Sterile 96-channel reservoirs (200 mL volume for pipetting system) (Mettler Toledo, catalog number: 17012605)

9. Breathe-easy foil (gas-permeable sealing membrane) (Sigma-Aldrich, catalog number: Z380059)

10. Transparent flat-bottom 96-well culture plates with lids for the inoculation of the bacteria in the treatment solution (200 μL volume) (Corning, catalog number: 3595)

## Equipment

**Figure 1.**
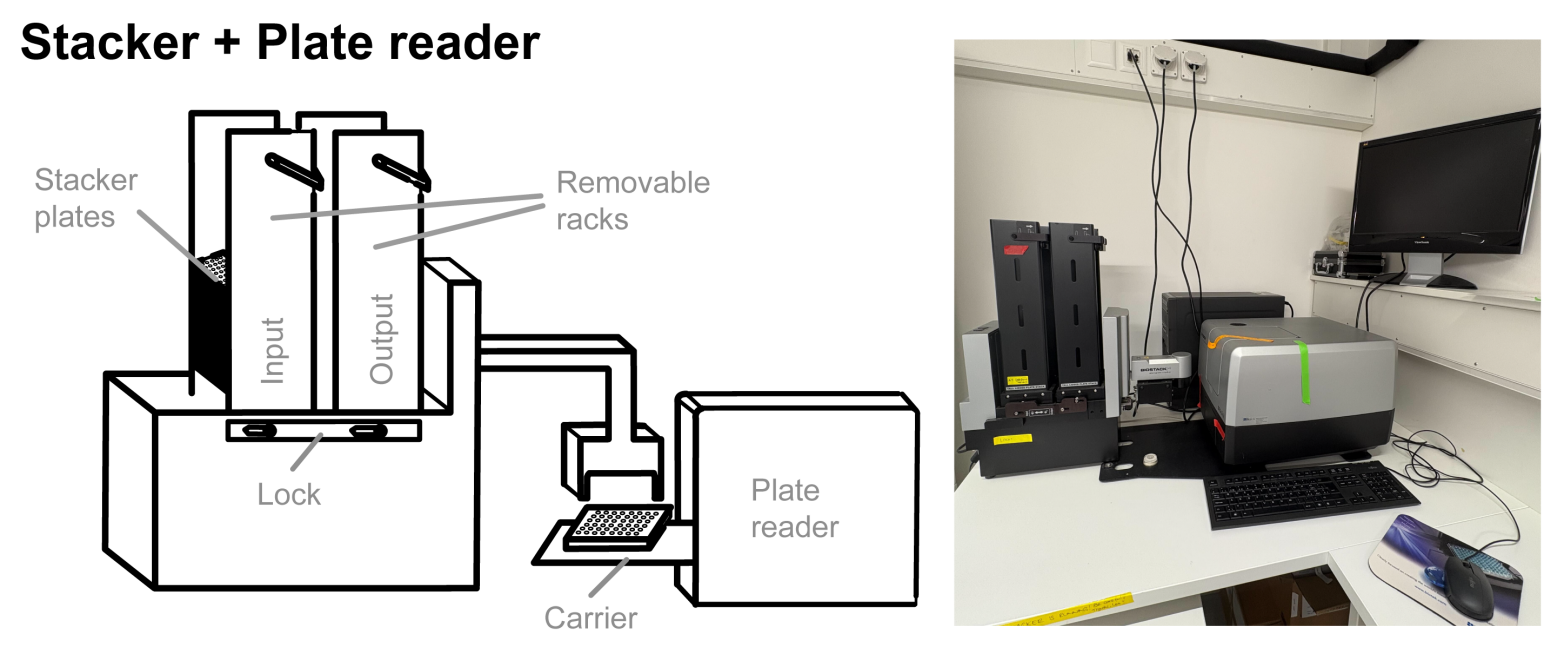
Stacker connected to a plate reader. The stacker connected to the plate reader handles up to 25 plates automatically. Plates are inserted into the input rack; then, a crawler moves the plate to the front, and the robotic arm removes the lid and places the plate onto the carrier. The carrier then inserts the plate into the plate reader, where the plate is shaken, and the optical density is measured. After the measurement, the plate is moved back, and the lid is added again and placed on the bottom of the output rack. When all plates are measured, they are automatically restacked to the input rack, and the process is repeated, allowing time series assays. The illustration (left) and photograph (right) show the stacker connected to the plate reader. A metal base plate fixes the distance between the two instruments.

A microplate stacker is a laboratory instrument that automatically stores and (un)loads multiple microplates (e.g., 96-well plates) into analytical instruments such as plate readers and can also handle lids if required. Combining such a stacker with a plate reader permits programming unattended time series assays, which greatly enhances the throughput. **Figure 1** illustrates a possible stacker system combined with a plate reader.

1. Stacker (Agilent Technologies, model: BioStack 4)

2. Plate reader (Agilent Technologies, model: Synergy H1)

3. 96-channel pipetting system (Mettler Toledo, model: Liquidator 96^TM^)

4. Incubator (28 °C) for cultivation of bacteria on agar plates (Infors-HT, Ecotron)

5. Shaking incubator (28 °C) for cultivation of bacteria in liquid cultures (Infors-HT, Ecotron)

6. Sterile bench equipped with a UV light lamp (berner, model: Claire-Neo)

## Software and datasets

1. Software to control plate reader and stacker: Gen5 (Agilent Technologies, version 3.09.07)

2. R statistical software [24] (version 4.2.2) with packages Tidyverse [25], emmeans [26], MESS [27], and dr4pl [28]

3. All data and code are available at GitHub (https://github.com/PMI-Basel/Thoenen_et_al_HT_Chem_Pheno_bacteria)

## Procedure

We describe the method based on an exemplary stacker run that comprises the screening of 60 bacterial isolates, including no-bacteria controls, testing them against one compound at seven concentration levels plus a solvent control. This run contains three replicates for each bacterial isolate, compound, and concentration combination. We recommend performing at least 2–3 independent runs to assess bacterial sensitivity.

All procedures are carried out at room temperature in a sterile hood unless indicated otherwise. The stacker and the plate reader are not in a hood.

**A. Preparing bacteria**

*Note: The exemplary stacker run that we describe here screens 60 bacterial isolate strains (BIS) using three technical replicates and includes no-bacteria controls (NBCs) to test for eventual contaminations during the stacker run.*

1. Prepare half-strength TSB as the liquid culture medium (15 g of TSB powder in 1 L of deionized water). A bottle of 250 mL is needed to prepare the preculture plates used in step A7 (3.75 g in 250 mL). Sterilize by autoclaving for 20 min at 121 °C. Store at room temperature until use.

2. Prepare full TSB media for agar plates (TSA) (30 g of TSB powder and 15 g of agar per L of deionized water); 60 strains require 1.5 L of TSA (60 × 25 mL) prepared in two bottles (used in step A3; each bottle with 22.5 g of TSB and 11.25 g of agar in 750 mL). Sterilize by autoclaving for 20 min at 121 °C. Ideally, pour the plates when the medium is still warm.

3. Pour approximately 25 mL of TSA into a 9 cm Ø petri dish. Prepare 60 plates. Let the plates dry for approximately 15 min with open lids in the sterile hood.

4. Inoculate the TSA plates with bacteria from glycerol stocks. To avoid thawing the stocks, scratch a bit of cells with a sterile inoculation loop from the glycerol stock tubes. Streak out the bacteria from the stock on the plate, allow to dry any eventual glycerol, and seal the plate with parafilm. Repeat for all 60 strains.

5. Incubate the plates at 28 °C for 4–10 days.

*Note: The age of the bacterial strains on the agar plate used for the preculture is important and should be kept constant to obtain comparable results. They should be freshly growing with sufficient biomass and not too old.*

**Figure 2.**
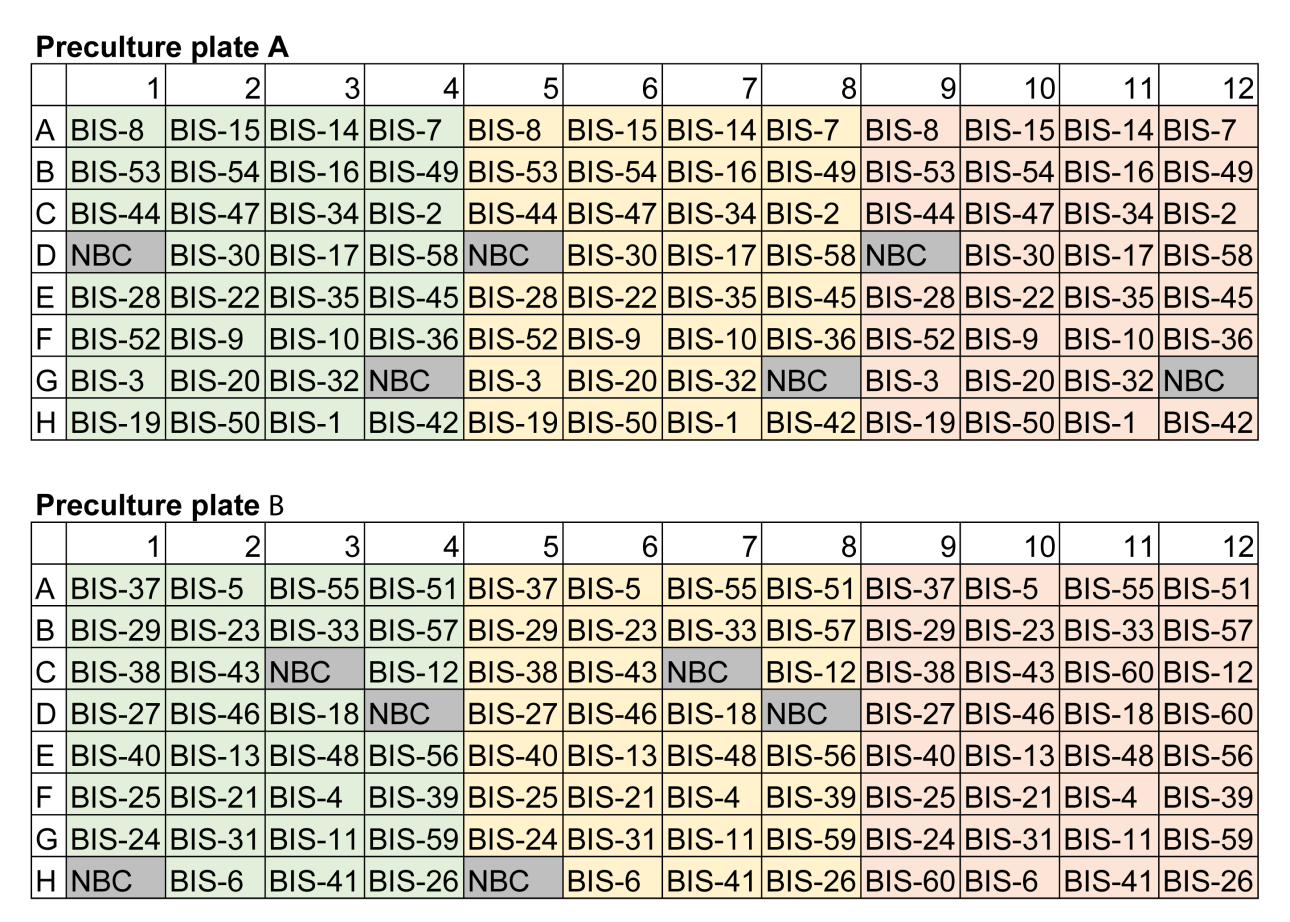
Example of a randomized plate layout. For the exemplary run, we organized 60 strains in two 96-well preculture plates (A and B). Each bacterial isolate (BIS) is replicated three times, and six no-bacteria controls (NBC) are organized in a randomized block layout. Save this predefined plate layout as meta information for the data analysis in R (section E).

6. Prepare preculture plates for the stacker run: Place two 2 mL round-bottom deep 96-well plates under UV light for 15 min to sterilize them. Distribute three replicates of each BIS according to a predefined scheme (see **Figure 2** for an example). Each preculture plate contains two NBCs of half-strength TSB medium without bacteria to test for eventual contaminations during the stacker run. Label plates with date, and plate A or B.

7. Fill a sterile 50 mL reagent reservoir with the prepared liquid half-strength TSB medium (step A1) and transfer 1 mL to each well using an 8-channel pipette.

8. With an inoculation needle, scratch a bit of bacterial cells off the agar plate. Dip the tip of the inoculation needle into the well and stir until the bacterial material comes off the tip. Slightly tilt the deep-well plate when doing this, to see better and avoid contamination by moving the inoculation needle over many other wells of the plate.

9. When finished, seal the plate with a breathe-easy foil (gas-permeable sealing membrane).

10. Incubate the deep-well plates in a laboratory shaker at 28 °C and 180 rpm for 4 days.

**B. Preparing treatments**

*Note: In this example, we test a single compound at 7 concentrations and include a solvent control (8 conditions in total).*

1. Prepare stock solution(s) of the chemical(s) to be tested. Dissolve the compound in an appropriate solvent in a 100× concentration (see General notes). For typical stacker runs, we use 500 mM. Filter-sterilize the 100× stock solution using a sterile, single-use 0.20 μm pore filter.

*Note: The choice of pore filters for sterilization depends on the solvent (should not affect the filter material) and the chemical compounds (should not adhere to the filter material).*

2. Prepare filter-sterilized solvent (>3 mL) for the controls.

3. Prepare half-strength TSB as detailed above. A bottle of 500 mL is needed to prepare the treatment solutions in the next step (7.5 g in 500 mL).

4. Prepare 8 × 50 mL centrifugation tubes labeled with the concentration to be tested and add 45 mL of the prepared half-strength TSB. Keep the remaining TSB in the bottle for step C1.

5. Add the compound from the 100× stock solution (500 mM) to reach the desired concentrations. A possible range of concentrations includes 0, 125, 250, 500, 750, 1,250, 2,500, and 5,000 μM. In the exemplary stacker run, the highest concentration (5,000 µM) corresponds to 450 μL of the stock solution to be added to the 45 mL of TSB. Prepare the other concentrations accordingly while keeping the solvent concentrations constant (in our example, each treatment solution contains 450 μL of solvent, 1% of the treatment solution). Prepare the treatment solutions immediately before starting the stacker run. It is not recommended to prepare them in advance and store them, due to the potential degradation of the compounds at room temperature.

**C. Assay setup**

The exemplary stacker run that we describe here requires setting up a total of 16 plates. The replicates of the 60 bacterial strains are distributed over 2 plates × 8 treatments. An additional *media control plate* containing only bacterial growth medium is utilized as contamination control. Additionally, we use empty stacker plates at the top and bottom of the stack as technical controls for handling by the instrument and to reduce eventual evaporation from sample plates. Hence, the exemplary stacker run contains a total of 19 plates. Label the stacker plates with chemical, concentration, and bacterial preculture plate A or B. Hence, there are two *stacker plates* per chemical and/or concentration.

**Figure 3.**
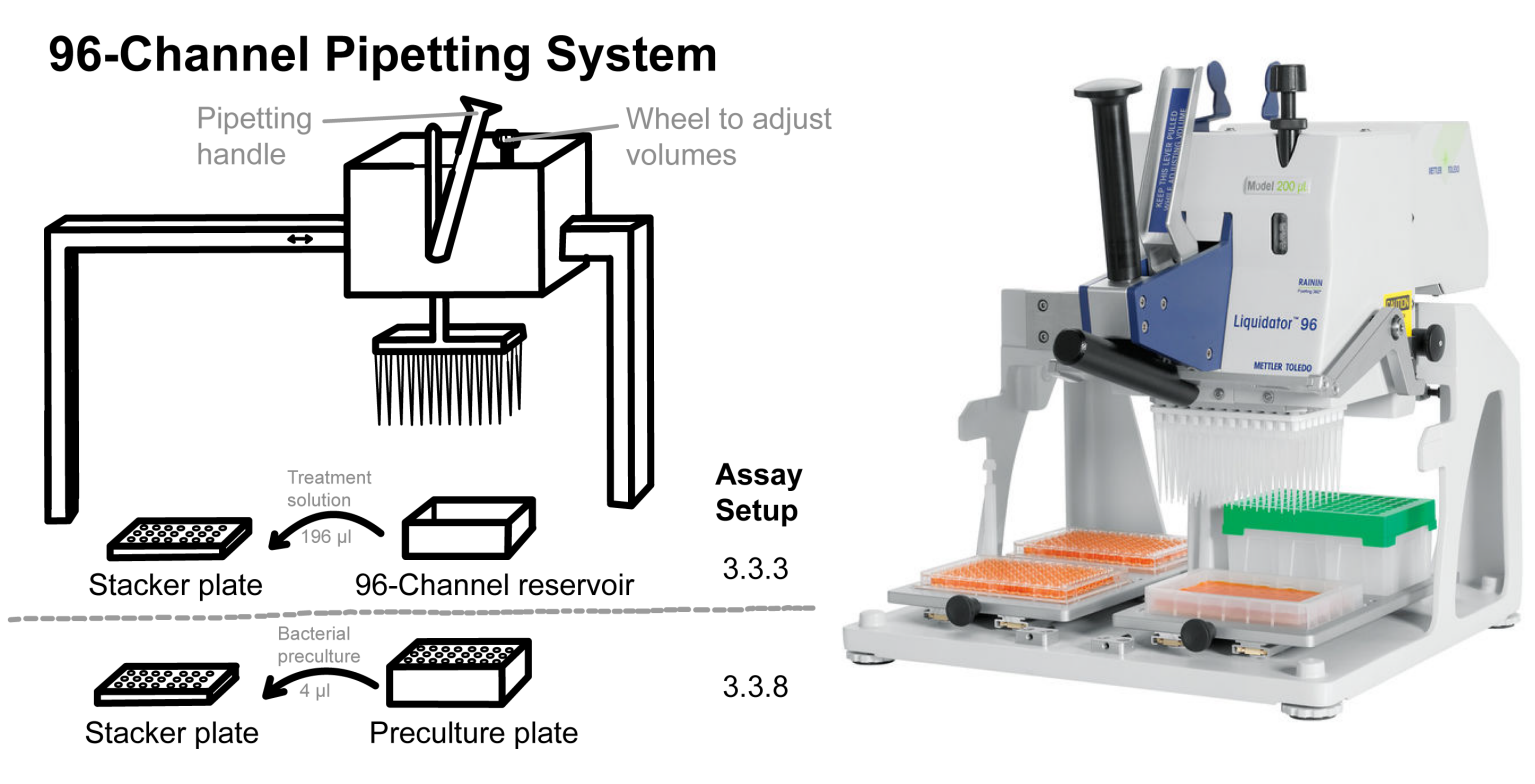
The 96-channel pipette system. Exact volumes can be transferred simultaneously from a reservoir or a preculture plate to all 96 wells of a stacker plate using such a pipetting system. Using this system, 196 μL of medium (step C3) or 4 μL of bacterial culture (step C8) is transferred to the stacker plate. The illustration (left) and the photograph (right) show a 96-channel pipetting system (the photograph of the Liquidator 96™ was taken from the website of Mettler Toledo).

Pipetting is greatly facilitated using a 96-channel pipetting system (see **Figure 3** for an illustration) that allows transfer of volumes ranging between 4 and 200 μL. Of course, the assay can be assembled using (automatic) 8- or 12-channel pipettes. However, in our experience, this was still much more time-consuming (a whole day for the exemplary assay) compared to the 96-channel pipetting system (approximately 3 h for the exemplary assay). Such a pipetting system helped us greatly reduce the time in the manual setup of the plates (see General notes).

1. Fill the sterile 96-channel reservoir with half-strength TSB (from the bottle of step B4) and prepare the media control plate. Add 200 μL of half-strength TSB to each well using the 96-channel pipetting system. Discard the remaining TSB from the reservoir, trying to remove as much as possible, without touching or otherwise contaminating it.

*Note: To reduce waste, the 200 mL reservoirs can be cleaned after each run by whipping with 70% ethanol and sterilizing by UV light for 15 min prior to setting up the new experiment.*

2. Next, assemble the stacker plates by adding the treatment solution (as prepared in step B5). First, pour the treatment solution with the lowest concentration into the reservoir.

3. Using the 96-channel pipetting system, fill 196 μL of treatment solution into each well of the stacker plate A. Repeat this step to fill the stacker plate B.

4. Fill the reservoir with the next concentration of treatment solution (as prepared in step B5). Repeat the step above and continue filling the stacker plates with their corresponding treatment concentrations. Stick to the order from the lowest to the highest compound concentration.

5. Discard used pipette tips.

6. Prepare two stacks, one with all concentrations of the A stacker plates and another with the B stacker plates.

7. Change the pipetting volume on the 96-channel pipetting system to 4 μL and add new tips.

8. Take the bacterial preculture plates with the bacteria from step A10 from the shaker. Start with the plate A and carefully remove the breathe-easy foil. Pipette 4 μL of preculture into each of the eight A stacker plates; again, start with the plate with the lowest chemical concentration and proceed to the highest concentration.

***Critical*:** When pipetting the bacterial precultures, be careful to dip the tips only halfway into the deep wells, not to stir the bacterial culture, and to avoid cross-contamination. Do not mix the bacterial cultures with the 96-channel pipetting system. Discard the used pipette tips.

9. Changing to the B preculture plate, add new tips to the 96-channel pipetting system. Repeat the step above to load the bacteria of the B plate to the B stacker plates.

10. Stack the plates onto each other according to the predefined order into a label list (save as *labellist.xlsx*; this information will be used in step E1). Do not forget to add the *media control plate*. Add empty stacker plates to the bottom and the top of the stack. Keep in mind that the lowest plate will be read first. Make sure that all plates of the stack are uniformly oriented with the A1 well in the top-left corner.

11. Carefully load the plates onto the input rack of the stacker, respecting the loading orientation (A1 well in the corresponding corner of the rack). Load the rack to the stacker and close the locks before starting the run.

12. Discard all leftovers of liquid bacterial material according to biosafety guidelines.

**D. Stacker run**

*Note: The exemplary stacker run consists of regular measurements of OD_600_ with prior shaking during the 68 h experiment. As a model, we provide the instrument protocol (GrowthCurveOD600.prt) from the exemplary stacker run on our GitHub.*

1. Make sure that both the plate reader and stacker are turned on and are connected.

2. Start the Gen5 program. Do not start the run immediately; check the protocol first. Click on *protocol*, then *procedure* to inspect the following settings: *Read* ≤ 600 nm, *Shake* ≤ linear for 2:00. The parameter *Shake* sets how and for how long each plate is agitated before the measurements. The protocol needs to be adjusted if the assay is extended to additional readouts and purposes (see General notes). The stacker runs continuously, and plates are measured in constant intervals depending on the number of plates in the run. Intervals between the measurements may be prolonged prior to the run in the protocol settings.

3. Click the *Start* button on the bottom right of the screen: Tick *use stacker and plates have lids*; do not tick *leave lids on during read*. The stacker will remove the lids immediately before loading the plates onto the reader and place the lid back on when the plate comes out of the reader again.

*Note: The plates may be read without the lid. Here, we do not cover the plates with a breathe-easy foil since that may cause problems for the stacker to handle plates due to eventual blockage.*

4. Start the stacker.

5. Let the stacker run for the desired amount of time. We typically use 68 h; up to this assay length, we did not find contamination in the NBCs and media control plates. During those 68 h, it is recommended to periodically check that there are no blockages and that the data are recorded.

*Note: The length of the run can be adjusted according to the growth rates of the tested bacteria. Attention is required when testing slow-growing cultures, as an extended assay length increases the risk of environmental contamination. Very careful checks of NBCs and media control plates are needed. Also, extended assays suffer from evaporation of the liquid media. In general, we do not recommend assays longer than 72 h.*

6. After the desired amount of time, stop the run by pressing the *Stop* button. If possible, stop a run after all plates of a stack have been measured.

7. Save the data by selecting all plates and clicking *export*. The Gen5 software exports your data as an Excel file that can be saved to your computer.

8. Discard all plates with the liquid bacterial material according to guidelines.

**E. Data analysis in R**

**Figure 4.**
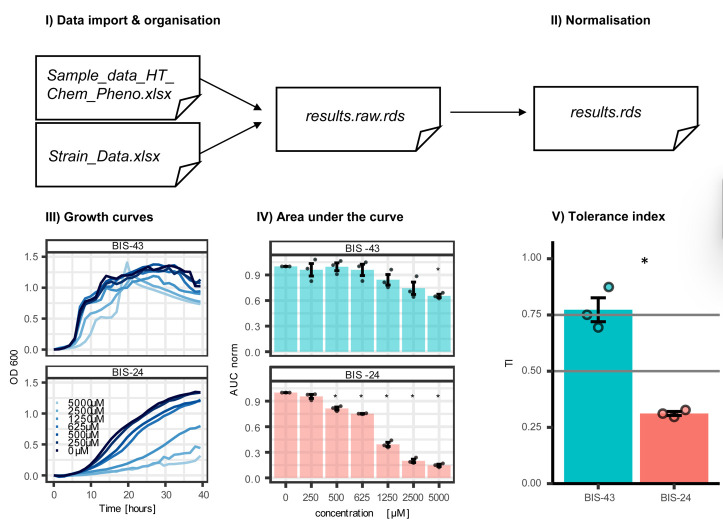
Data analysis workflow. I) Data analysis starts with importing the raw growth data from the plate reader into R and combining it with the metainformation of the experiment. II) The growth data is normalized for a given strain by correcting for differences in input densities (OD_600_), resulting in the actual growth curves based on optical density. III) Optical density data are represented as growth curves. IV) Areas under the curve (AUC) are calculated to approximate the growth of a bacterial isolate (BIS) in each concentration. V) The tolerance index (TI) is deduced from the AUC values of a strain across the tested concentrations. The TI corresponds to a measure of tolerance of a given strain to a given chemical. The analysis is exemplified based on a representative tolerant strain (BIS-43) and a representative susceptible strain (BIS-24). Bar graphs (IV and V) display means ± SE of n = 3 with individual datapoints together with appropriate statistics (t-test, significance indicated with an asterisk).

*Note: We summarize the different steps of the data analysis from data import to calculating the tolerance index (TI) in* ***Figure 4***.

For exemplary data analysis, we provide the following files on our GitHub: a model R script and its output (Script: *HT_Chem_Pheno.rmd;* Output: *HT_Chem_Pheno.html*) together with meta information (*Strain_Data.xlsx* and *labellist.xlsx*) and raw growth data (*Sample_data_HT_Chem_Pheno.xlsx*) from the exemplary stacker run.

1. Data import and organization: The *Strain_Data.xlsx* file contains the well positions of the replicates in the plates and further metadata like the bacteria’s taxonomy. Load the exported raw growth data (*Sample_data_HT_Chem_Pheno.xlsx*). Then, load the *labellist.xlsx* containing the order of the stacker plates during the run (see step C10) with their corresponding metadata of preculture plate (A or B), medium, compound, concentration, and stacker run. If multiple stacker runs are analyzed, combine their raw growth data.

2. Normalization: Growth data is corrected for differences in densities (OD_600_) at the start of the run. The optical density at the first measurement is subtracted from all measurements at the following time points. Further, the maximal optical density of each well is calculated and plotted, which allows to detect strains with poor growth. These strains can be manually excluded from the analysis.

3. Growth curves: The provided script contains a plotting function to inspect the growth of the single strains per well or per treatment across the time course. This allows, again, to detect strains or wells with poor growth.

4. Area under the curve: Using the function *auc()* from the MESS package [27], the AUC is calculated. It reflects the overall growth of a BIS in each well. The AUC parameter accounts for variability in growth resulting from, e.g., clumping or biofilm formation. Next, the AUC of a given concentration is normalized to the AUC of the control treatment (concentration 0, solvent). The provided script contains a plotting function to visualize and compare the normalized AUC values (AUC norm) between strains across all concentrations.

5. Tolerance index: Using the *auc()* function again, the TI is calculated across the AUC norm values for each strain. This TI represents the tolerance of a strain across all tested concentrations. We consider strains with TI values > 0.75 as tolerant, TI values 0.5–0.75 as intermediate, and values <0.5 as susceptible to the tested chemical. In contrast to defining medium inhibitory concentrations (IC50), the TI method allows tolerance comparisons among strains even if some of them are not inhibited at the highest tested concentration.

*Note: The provided R script contains a function to calculate the inhibitory concentration 50 (IC50): IC50 is where growth is inhibited to 50% and can be estimated from the AUC dataset. Differences in IC50 between isolates typically indicate differences in tolerance; lower IC50 values indicate a higher tolerance to the tested compound. The IC50 values are log10-transformed and can be transformed back to the original scale of concentrations* via *10^(IC50). Determination of IC50 is only meaningful if all compared strains are sensitive to the tested compound. IC50 cannot be calculated for strains that are not inhibited at the highest tested concentration.*

## Validation of protocol

This protocol (or parts of it) has been used and validated in the following research article(s):

- Schandry et al. [20] Plant-derived benzoxazinoids act as antibiotics and shape bacterial communities. *bioRxiv* (Figure 1).
- Thoenen et al. [11] Bacterial Tolerance to Host-Exuded Specialized Metabolites Structures the Maize Root Microbiome. *Proceedings of the National Academy of Sciences* (Figure 2A–C; Figure 3A Figures S3–S11).
- Thoenen et al. [21] The Lactonase BxdA Mediates Metabolic Specialisation of Maize Root Bacteria to Benzoxazinoids. *Nature Communications* (Figure 2D; Figure 5E; Figures S6 and S11).
- Rouyer et al. [22] Plant specialised metabolites modulate the molecular signatures of host-bacteria and bacteria-bacteria interactions. *bioRxiv* (Figure 1a).
- Thoenen et al. [23] Synthetic Communities of Maize Root Bacteria Interact and Redirect Benzoxazinoid Metabolization. *mSphere* (Figures S2 and S5).

## General notes and troubleshooting

**General notes**

1. Choice of bacteria: This approach is suitable for aerobic bacteria that grow well in the given experimental conditions of the stacker assay (e.g., temperature, media). Bacteria that form strong biofilms, clump, or do not grow as homogeneous liquid cultures (e.g., *Streptomyces*) are not suitable for the assay, since OD_600_ measurements do not reflect their growth correctly.

2. Choice of media: This assay is scalable to various bacterial growth media, depending on the preferences of diverse bacteria. Half-strength TSB proved to be a suitable medium for testing plant-associated bacteria because it works for diverse taxa and because little contamination occurred during stacker runs. Minimal media allow testing if a strain can use a certain compound as the carbon source. Richer media or media favoring the growth of environmental- or human-associated bacteria may be more prone to contamination.

3. Choice of chemicals: With this system, a broad range of compounds can be tested, including specialized metabolites of plants, antibiotics, and many more, such as pharmaceuticals, metals, biocides, and pesticides. For the compound(s) to be tested, chemical stability in the test conditions is crucial. Compounds that degrade under the experimental conditions (e.g., temperature, light, pH, oxygen, medium) cannot be tested. Also, compounds with an absorbance at ~OD_600_ are not suitable for the assay since they interfere with the quantification of bacterial growth. A compound’s solvent is optimal if it exerts minimal toxicity on the bacteria and at the same time permits dissolving high concentrations of the compound of interest. DMSO is suitable for dissolving many compounds with low solubility, as it only minimally interferes with bacterial growth.

4. Choice of measurement: Besides measuring OD_600_, the reader may be used for other additional measurements like absorbance and/or fluorescence at different wavelengths, allowing to extend the assay to additional readouts and purposes. Of note, the inclusion of pH-sensitive dyes (e.g., bromophenol blue) may be problematic since several bacteria change the pH of their medium during growth.

5. Manual setup: The main advantage of the approach is the automated reading of multiple plates (e.g., up to 25 plates). A limitation remains that the inoculation of individual wells is performed manually; this remains the most time-consuming step in the workflow.

## References

[1] Canarini A., Kaiser C., Merchant A., Richter A. and Wanek W. (2019). Root Exudation of Primary Metabolites: Mechanisms and Their Roles in Plant Responses to Environmental Stimuli. Front Plant Sci. 10: e00157. 10.3389/fpls.2019 .00157 PMC6407669 30881364

[2] Erb M. and Kliebenstein D. J. (2020). Plant Secondary Metabolites as Defenses, Regulators, and Primary Metabolites: The Blurred Functional Trichotomy. Plant Physiol. 184(1): 39 52 52. 10.1104/pp.20 .00433 32636341 PMC7479915

[3] Jacoby R. P., Koprivova A. and Kopriva S. (2020). Pinpointing secondary metabolites that shape the composition and function of the plant microbiome. J Exp Bot. 72(1): 57 69 69. 10.1093/jxb/eraa424 PMC7816845 32995888

[4] Guo B., Zhang Y., Li S., Lai T., Yang L., Chen J. and Ding W. (2016). Extract from Maize(Zea mays L.): Antibacterial Activity of DIMBOA and Its Derivatives against Ralstonia solanacearum. Molecules. 21(10): 1397 10.3390/molecules21101397 27775575 PMC6273367

[5] Huang A. C., Jiang T., Liu Y. X., Bai Y. C., Reed J., Qu B., Goossens A., Nützmann H. W., Bai Y., Osbourn A., .(2019). A specialized metabolic network selectively modulates *Arabidopsis* root microbiota. Science. 364(6440): eaau6389. https://doi.org/10.1126/science.aau6389 31073042

[6] Neal A. L., Ahmad S., Gordon-Weeks R. and Ton J. (2012). Benzoxazinoids in Root Exudates of Maize Attract Pseudomonas putida to the Rhizosphere. PLoS One. 7(4): e35498. 10.1371/journal.pone .0035498 PMC3335876 22545111

[7] Stassen M. J., Hsu S. H., Pieterse C. M. and Stringlis I. A. (2021). Coumarin Communication Along the Microbiome–Root–Shoot Axis. Trends Plant Sci. 26(2): 169 183 183. 10.1016/j.tplants .2020.09.008 33023832

[8] Sugiyama A. (2021). Flavonoids and saponins in plant rhizospheres: roles, dynamics, and the potential for agriculture. Biosci, Biotechnol, Biochem. 85(9): 1919 1931 1931. 10.1093/bbb/zbab106 34113972

[9] Harbort C. J., Hashimoto M., Inoue H., Niu Y., Guan R., Rombolà A. D., Kopriva S., Voges M. J., Sattely E. S., Garrido-Oter R., .(2020). Root-Secreted Coumarins and the Microbiota Interact to Improve Iron Nutrition in Arabidopsis. Cell Host& Microbe 28(6): 825–837.e6. 10.1016/j.chom .2020.09.006 PMC7738756 33027611

[10] Stringlis I. A., Yu K., Feussner K., de Jonge R., Van Bentum S., Van Verk M. C., Berendsen R. L., Bakker P. A. H. M., Feussner I., Pieterse C. M. J., .(2018). MYB72-dependent coumarin exudation shapes root microbiome assembly to promote plant health. Proc Natl Acad Sci USA. 115(22): e1722335115. https://doi.org/10.1073/pnas.1722335115 PMC5984513 29686086

[11] Thoenen L., Giroud C., Kreuzer M., Waelchli J., Gfeller V., Deslandes-Hérold G., Mateo P., Robert C. A. M., Ahrens C. H., Rubio-Somoza I., .(2023). Bacterial tolerance to host-exuded specialized metabolites structures the maize root microbiome. Proc Natl Acad Sci USA. 120(44): e2310134120. https://doi.org/10.1073/pnas.2310134120 PMC10622871 37878725

[12] Gajic I., Kabic J., Kekic D., Jovicevic M., Milenkovic M., Mitic Culafic D., Trudic A., Ranin L. and Opavski N. (2022). Antimicrobial Susceptibility Testing: A Comprehensive Review of Currently Used Methods. Antibiotics. 11(4): 427 10.3390/antibiotics11040427 35453179 PMC9024665

[13] Bai Y., Müller D. B., Srinivas G., Garrido-Oter R., Potthoff E., Rott M., Dombrowski N., Münch P. C., Spaepen S., Remus-Emsermann M., .(2015). Functional overlap of the Arabidopsis leaf and root microbiota. Nature. 528(7582): 364 369 369. 10.1038/nature16192 26633631

[14] Beirinckx S., Viaene T., Haegeman A., Debode J., Amery F., Vandenabeele S., Nelissen H., Inzé D., Tito R., Raes J., .(2020). Tapping into the maize root microbiome to identify bacteria that promote growth under chilling conditions. Microbiome. 8(1): e1186/s40168–020–00833–w. 10.1186/s40168-020-00833-w PMC7166315 32305066

[15] Hartman K., van der Heijden M. G., Roussely-Provent V., Walser J. C. and Schlaeppi K. (2017). Deciphering composition and function of the root microbiome of a legume plant. Microbiome. 5(1): e1186/s40168–016–0220–z. 10.1186/s40168-016-0220-z PMC5240445 28095877

[16] Niu B., Paulson J. N., Zheng X. and Kolter R. (2017). Simplified and representative bacterial community of maize roots. Proc Natl Acad Sci USA. 114(12): e1616148114. https://doi.org/10.1073/pnas.1616148114 PMC5373366 28275097

[17] Wippel K., Tao K., Niu Y., Zgadzaj R., Kiel N., Guan R., Dahms E., Zhang P., Jensen D. B., Logemann E., .(2021). Host preference and invasiveness of commensal bacteria in the Lotus and Arabidopsis root microbiota. Nat Microbiol. 6(9): 1150 1162 1162. 10.1038/s41564-021-00941-9 34312531 PMC8387241

[18] Zhang J., Liu Y. X., Zhang N., Hu B., Jin T., Xu H., Qin Y., Yan P., Zhang X., Guo X., .(2019). NRT1.1B is associated with root microbiota composition and nitrogen use in field-grown rice. Nat Biotechnol. 37(6): 676 684 684. 10.1038/s41587-019-0104-4 31036930

[19] Maier L., Pruteanu M., Kuhn M., Zeller G., Telzerow A., Anderson E. E., Brochado A. R., Fernandez K. C., Dose H., Mori H., .(2018). Extensive impact of non-antibiotic drugs on human gut bacteria. Nature. 555(7698): 623 628 628. 10.1038/nature25979 29555994 PMC6108420

[20] Schandry N., Jandrasits K., Garrido-Oter R. and Becker C. (2021). Plant-derived benzoxazinoids act as antibiotics and shape bacterial communities. bioRxiv. 10.1101/2021.01.12.425818

[21] Thoenen L., Kreuzer M., Pestalozzi C., Florean M., Mateo P., Züst T., Wei A., Giroud C., Rouyer L., Gfeller V., .(2024). The lactonase BxdA mediates metabolic specialisation of maize root bacteria to benzoxazinoids. Nat Commun. 15(1): e1038/s41467–024–49643–w. 10.1038/s41467-024-49643-w PMC11297187 39095376

[22] Rouyer L., Becker C. and Schandry N. (2025). Plant specialised metabolites modulate the molecular signatures of host-bacteria and bacteria-bacteria interactions. bioRxiv. https://doi.org/10.1101/2025.06.06.658260

[23] Thoenen L., Pestalozzi C., Zuest T., Kreuzer M., Mateo P., Karasawa M., Deslandes G., Robert C. A., Bruggmann R., Erb M., .(2025). Synthetic communities of maize root bacteria interact and redirect benzoxazinoid metabolization. mSphere. 10(9): e00159–25. https://doi.org/10.1128/msphere.00159-25 PMC12483121 40853002

[24] R Core Team(2025). R: A Language and Environment for Statistical Computing. Vienna, Austria: R Foundation for Statistical Computing. Retrieved from https://www.R-project.org/

[25] Wickham H., Averick M., Bryan J., Chang W., McGowan L., François R., Grolemund G., Hayes A., Henry L., Hester J., .(2019). Welcome to the Tidyverse. J open source softw. 4(43): 1686 10.21105/joss.01686

[26] Lenth R., Sigmann H., Love J., Buerkner P. and Herve M. (2019). emmeans: Estimated Marginal Means, aka Least-Squares Means. 10.32614/cran.package.emmeans

[27] Ekstrøm C. (2016). MESS: Miscellaneous Esoteric Statistical Scripts. https://github.com/ekstroem/mess.

[28] Ritz C., Baty F., Streibig J. C. and Gerhard D. (2015). Dose-Response Analysis Using R. PLoS One. 10(12): e0146021. 10.1371/journal.pone .0146021 PMC4696819 26717316

